# Epoxyeicosatrienoic Acid Analog EET-A Blunts Development of Lupus Nephritis in Mice

**DOI:** 10.3389/fphar.2019.00512

**Published:** 2019-05-10

**Authors:** Md. Abdul Hye Khan, Anna Stavniichuk, Mohammad Abdul Sattar, John R. Falck, John D. Imig

**Affiliations:** ^1^Department of Pharmacology and Toxicology, Medical College of Wisconsin, Milwaukee, WI, United States; ^2^Department of Biochemistry, UT Southwestern Medical Center, Dallas, TX, United States

**Keywords:** lupus nephritis, EET analog, inflammation, chemokine, small lipid mediators, mouse

## Abstract

Systemic lupus erythematosus (SLE) is a chronic autoimmune inflammatory disorder that causes life threatening renal disease and current therapies are limited with serious side-effects. CYP epoxygenase metabolites of arachidonic acid epoxyeicosatrienoic acids (EETs) demonstrate strong anti-inflammatory and kidney protective actions. We investigated the ability of an orally active EET analog, EET-A to prevent kidney injury in a mouse SLE model. Twenty-weeks old female NZBWF1 (SLE) and age-matched NZW/LacJ (Non SLE) were treated with vehicle or EET-A (10 mg/kg/d, p.o.) for 14 weeks and urine and kidney tissues were collected at the end of the protocol. SLE mice demonstrated marked renal chemotaxis with 30–60% higher renal mRNA expression of CXC chemokine receptors (CXCR) and CXC chemokines (CXCL) compared to Non SLE mice. In SLE mice, the elevated chemotaxis is associated with 5-15-fold increase in cytokine mRNA expression and elevated inflammatory cell infiltration in the kidney. SLE mice also had elevated BUN, serum creatinine, proteinuria, and renal fibrosis. Interestingly, EET-A treatment markedly diminished renal CXCR and CXCL renal mRNA expression in SLE mice. EET-A treatment also reduced renal TNF-α, IL-6, IL-1β, and IFN-γ mRNA expression by 70–80% in SLE mice. Along with reductions in renal chemokine and cytokine mRNA expression, EET-A reduced renal immune cell infiltration, BUN, serum creatinine, proteinuria and renal fibrosis in SLE mice. Overall, we demonstrate that an orally active EET analog, EET-A prevents renal injury in a mouse model of SLE by reducing inflammation.

## Introduction

Systemic lupus erythematosus (SLE) is an autoimmune disease characterized by abnormality in the immune system. In SLE, almost all organs in the body can be involved and clinical presentation of SLE can range from mild to severe depending on the affected organ. Involvement of the kidney is termed as lupus nephritis (LN) which affects up to 60% of the SLE patients and remains the leading cause of morbidity and mortality in SLE (Lu et al., 2012). The etiopathology of SLE as well as LN are still not fully understood and this lack of knowledge limits therapeutic options for SLE and LN (Lech and Anders, 2013).

The current therapy of LN involves the use of corticosteroids and the alkylating agent cyclophosphamide. Indeed, a combination of these agents is now considered as the standard of care for SLE and LN and improves patient survival rate. However, serious side effects that occur with immunosuppression are common with these therapies and are associated with significant morbidity (Contreras et al., 2004; Gilkeson, 2015). As such, there is a need to develop novel therapies for LN treatment that will improve long-term renal outcomes with minimum treatment-related toxicity. Unfortunately, the success in developing novel and safer therapy for LN is still unsatisfactory and the current therapeutic approaches still depend on high-dose corticosteroids combined with broad-spectrum immunosuppressive agents (Gilkeson, 2015; Jordan and D’Cruz, 2016; Dall’Era, 2017). This lack of novel LN therapies clearly indicates an unmet need for research and development of novel LN treatment options.

One possible approach to develop novel LN therapy can be the use of epoxyeicosatrienoic acids (EETs), the cytochrome P450 epoxygenase metabolites of arachidonic acid. Several studies have demonstrated kidney protective actions for EETs and EET synthetic analogs in pre-clinical kidney disease models (Hye Khan et al., 2013, 2016; Yeboah et al., 2016). These studies determined that synthetic EET analogs reduce renal tubular and glomerular injuries (Hye Khan et al., 2013, 2016). EET analogs also demonstrated strong anti-fibrotic actions and prevented or treated kidney fibrosis in multiple pathologies that contribute to chronic kidney disease (Hye Khan et al., 2013, 2016; Khan et al., 2013; Skibba et al., 2017). We demonstrated that the biological actions of EETs and EET analogs have strong anti-inflammatory, anti-apoptotic and anti-oxidative actions that contribute to the kidney protective actions (Khan et al., 2013; Hye Khan et al., 2013, 2016).

In the present study, we investigated the ability for an orally active EET analog, EET-A, to blunt the LN development in a mouse pre-clinical SLE model. We provide evidence that EET-A anti-inflammatory actions can prevent renal injury development in SLE mice.

## Materials and Methods

### Chemicals

The EET analog, EET-A, was designed and synthesized in the laboratory of JF (Department of Biochemistry, University of Texas Southwestern Medical Center, Dallas, TX). Unless mentioned otherwise, all chemicals used in the current study were obtained from Sigma Aldrich (St Louis, MO, United States).

### Animal Experiments

Twenty weeks old female NZBWF1 (SLE) and NZW/LacJ (Non SLE) obtained from Jackson Laboratories (Bar Harbor, ME, United States) were used in this study. Mice had free access to food and water and were housed with 12/12h light-dark cycle in the Biomedical Resource Center at the Medical College of Wisconsin. The mice were divided into three groups based on their baseline systolic blood pressure measured by tail-cuff plethysmography. Non SLE NZW/LacJ mice received vehicle and the NZBWF1 SLE mice received either vehicle or EET-A (10 mg/kg/d) in the drinking water for 14 weeks. The dose of EET-A is based on previous experimental studies demonstrating that appropriate EET-A plasma concentrations were achieved (Khan et al., 2013). At the end of the experimental protocol, urine, and blood samples were collected followed by kidney tissue collection for biochemical, histopathological, and other analysis. Animal experiments were performed with the approval of the Medical College of Wisconsin Institutional Animal Care and Use Committee and in accordance with National Institutes of Health guidelines.

### Biochemical Analysis

Urine samples were collected at the end of the experimental protocol by placing mice in metabolic cages for 24 h. Blood samples were collected from abdominal aorta under isoflurane anesthesia followed by killing of the animal with anesthetic overdose and kidney tissue collection. Urine and serum samples were analyzed using a commercially available assay for creatinine from Cayman Chemicals (Ann Arbor, MI, United States). Urine albumin level was measured using a kit from Exocell (Philadelphia, PA, United States). Blood Urea Nitrogen (BUN) was measured using a colorimetric assay (Thermo Fisher Scientific, Waltham, MA, United States). Plasma anti-dsDNA antibodies were measured with a commercial ELISA (Alpha Diagnostic International, San Antonio, TX, United States). Urine albumin and creatinine data were used to calculate the ratio of urinary albumin and creatinine excretion, which is a measure of the kidney injury.

### Real-Time PCR Analysis

Renal mRNA expression for several chemokine and chemokine receptors associated with the pathophysiology of chronic inflammatory autoimmune disease were analyzed using real-time PCR (RT-PCR). RT-PCR analysis was carried for the renal mRNA expression of CXC motif chemokine ligand 9 (CXCL9), 10 (CXCL10), 13 (CXCL13) and 16 (CXCL16). We also analyzed the mRNA expression of CXC chemokine receptors 3 (CXCR3) and 4 (CXCL4). Renal mRNA expression of several cytokines namely tumor necrosis factor-α (TNF-α), interleukin 6 (IL-6), interleukin 1-β (IL-1β), and interferon gamma (IFN-γ) was determined. The primer sequence for the CXC chemokine, chemokine receptor and cytokines are provided in Table 1. Renal cortex was carefully dissected from sagittal kidney section using a dissecting microscope. Absence of any medullary tissue was confirmed using microscopic observation. Renal cortex was then homogenized for mRNA extraction and RT PCR analysis. In RT-PCR analysis, mRNA was prepared from kidney cortical tissue using RNeasy Mini Kit (QIAGEN, CA, United States) according to the manufacturer’s protocol. The quality and quantity of the mRNA samples were determined spectrophotometrically. Synthesis of cDNA was carried out from the mRNA samples using iScript^TM^ Select cDNA Synthesis Kit (Bio-Rad, Hercules, CA, United States). Expression of the genes was quantified by iScript One-Step RT-PCR Kit with SYBR green using the MyiQ^TM^ Single Color Real-Time PCR Detection System (Bio-Rad Laboratories, Hercules, CA, United States). All mRNA samples were run in triplicate and fold change in the target gene expression compared to the expression of control genes by comparative threshold cycle (*C*_t_) method. Target gene expression levels were determined by normalizing the *C*_t_ values to two control genes.

**Table 1:** Primer sequences of the target genes used in RT PCR analysis.

CXCR3	F- TCTCGTTTTCCCCATAATCG
	R- AGCCAAGCCATGTACCTTGA
CXCL9	F- CGAGGCACGATCCACTACAA
	R- GAGTCCGGATCTAGGCAGGT
CXCL10	F- ACTGCATCCATATCGATGAC
	R- TTCATCGTGGCAATGATCTC
CXCR4	F- GTTGCCATGGAACCGATCA
	R- TGCCGACTATGCCAGTCAAGA
CXCL13	F- CAGGCCACGGTATTCTGGA
	R- CAGGGGGCGTAACTTGAATC
CXCL16	F-CGTTGTCCATTCTTTATCAGGTTCC
	R- TTGCGCTCAAAGCAGTCCA
TNF-α	F- CGAGTGACAAGCCTGTAGCC
	R- GAGAACCTGGGAGTAGACAAGG
IL-6	F- TGTATGAACAACGATGATGCAC
	R- TGGTACTCCAGAAGACCAGAGG
IL-1β	F- AAGGAGAACCAAGCAACGAC
	R- AACTCTGCAGACTCAAACTCCAC
IFN-γ	F- AGCAAGGCGAAAAAGGATGC
	R- TCATTGAATGCTTGGCGCTG


### Histopathology

Histopathological analysis of renal fibrosis was done using 10% formalin fixed kidney tissue. The formalin fixed kidney tissues were paraffin-embedded, sectioned (5 μm), mounted on slides and stained with Picrosirius Red (PSR) stain (Alfa Aesar, Tewksbury, MA). PSR stained slides were examined for interstitial collagen at 200x magnification and the collagen positive fibrotic kidney area was calculated as an index of renal fibrosis using NIS Elements AR version 3.0 imaging software (Nikon instruments Inc., Melville, NY, United States). Renal fibrosis was scored in a blinded fashion by two observers as published previously (Hye Khan et al., 2016, 2018), and the scores were presented as a percentage area-fraction relative to the total area analyzed.

### Immunohistopathological Analysis

Deparaffinized kidney sections mounted on slides were re-hydrated followed by overnight incubation with rat anti-mouse CD43 antibody (1:100, BD Biosciences, San Jose, CA, United States). The slides were washed and incubated with biotinylated goat anti-rat secondary antibody (1:200, abcam, Cambridge, MA, United States) for 1 h. Slides were developed with avidin-biotin and HRP complex (Vectastain ABC Elite kit, Vector Laboratories, Burlingame, CA, United States) followed by counterstaining with hematoxylin. Stained histological sections were visualized at 400× magnification with a light microscope and analyzed by two observers in a blinded fashion using Nikon NIS Elements Software (Nikon Instruments Inc., Melville, NY, United States).

### Immunofluorescence Analysis

Formalin formalin-fixed and paraffin-embedded kidney sections (5 μm) were de-paraffinized, re-hydrated, and incubated with rodent declocker solution (Biocare Medical, Concord, CA, United States) at 95°C for antigen retrieval. Kidney sections were then immunostained with rat monoclonal anti-F4/80 antibody (1:100; abcam, Cambridge, MA, United States) to determine renal expression of F4/80 positive inflammatory cells. Goat anti-rat IgG H&L (Alexa Fluor^®^ 488) secondary antibody (Abcam, United States) was used for development with fluorescence quenching liquid (Vector Laboratories, United States). Immunostained sections were examined by Nikon 55i fluorescence with a green excitation (200× magnification) and digital images were taken for analysis using Nikon NIS Elements Software (Nikon Instruments Inc., United States). The number of glomerular F4/80 positive cells was determined in 100 glomeruli by two blinded researchers and expressed as the number of cells per glomerulus.

### Statistical Analysis

All data are expressed as mean ± S.E.M. In order to determine statistical difference between different experimental groups GraphPad Prism^®^ Version 4.0 software was used to carry out one-way ANOVA followed by Tukey’s *post hoc* test (GraphPad Software Inc., La Jolla, CA, United States).

## Results

### EET-A Treatment Reduces Blood Pressure and Body Weight Loss in SLE Mice

Systemic lupus erythematosus mice had significantly higher (*P* < 0.05) plasma anti-dsDNA antibody levels (63 ± 2 U/L, *n* = 10) compared Non SLE mice (2.3 ± 0.1 U/L, *n* = 10), and EET-A treatment did not affect plasma levels of anti-dsDNA antibodies in SLE mice (59 ± 4 U/L, *n* = 10).

Systolic blood pressure averaged 84 ± 11 mmHg (*n* = 10) in the Non SLE mice group, 94 ± 7 mmHg (*n* = 10) in the vehicle treated SLE mice group, and 96 ± 9 mmHg (*n* = 10) in EET-A treated SLE mice at the start of the experimental protocol. SLE mice systolic blood pressure was significantly increased (137 ± 10 mmHg, *n* = 10) compared to Non SLE mice (89 ± 8 mmHg, *P* < 0.05, *n* = 10) at the end of 14 week-experimental protocol. EET-A treatme nt to SLE mice significantly decreased systolic blood pressure (104 ± 7 mmHg, *n* = 10) compared to vehicle treated SLE mice.

Body weight averaged 28.0 ± 1.7 g (*n* = 10) in the Non SLE mice group, 28.3 ± 2.2 g (*n* = 10) in the vehicle treated SLE mice group, and 27.8 ± 3.0 g (*n* = 10) in EET-A treated SLE mice at the start of the experimental protocol. At the end of 14-week-experimental protocol, the SLE mice had a lower body weight (22.0 ± 1.3 g, *n* = 10) compared to Non SLE mice (41.7 ± 2.0 g, *P* < 0.05, *n* = 10) and EET-A treated mice (37.8 ± 1.2 g, *P* < 0.05, *n* = 10).

### EET-A Treatment Decreases Renal CXCL Chemokine and CXC Receptors in SLE Mice

**FIGURE 1:**
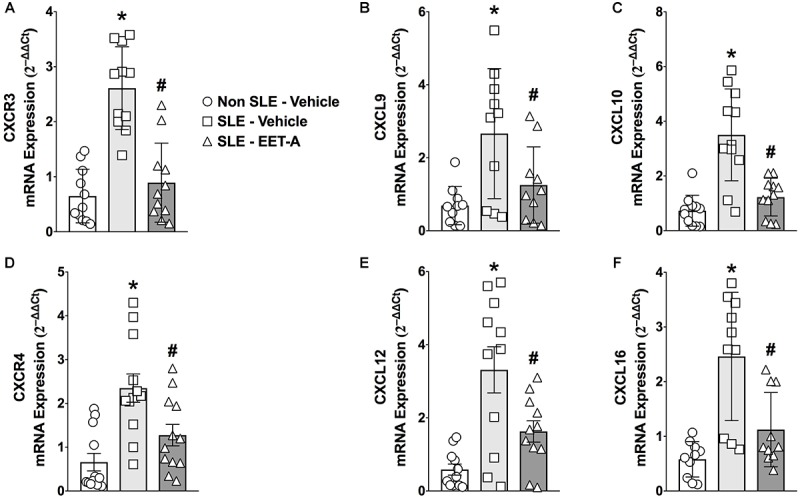
Orally active EET analog, EET-A, decreased renal cortical mRNA expression of CXC chemokine receptor CXCR3 **(A)** and its ligands CXCL 9 **(B)**, CXCL10 **(C)** in a mouse model of systemic lupus erythematosus (SLE). EET-A also reduced mRNA expression of CXC chemokine receptor CXCR4 **(D)** and CXC chemokines CXCL13 **(E)** and CXCL16 **(F)** in the kidney cortex of SLE mice. All data are expressed as Mean ± SEM, ^∗^*P* < 0.05 vs. Non SLE-Vehicle, ^#^*P* < 0.05 vs. SLE-Vehicle, *n* = 10–12/group. NZBWF1 (SLE) and NZW/LacJ (Non SLE).

Systemic lupus erythematosus mice had a 3 to 5-fold higher renal cortical mRNA expression of lymphocyte-specific CXC chemokines (CXCL9,10,13, and 16) and CXC receptors (CXCR3 and 4) that contribute to SLE pathophysiology (Figure 1). Interestingly, EET-A treatment for 14 weeks markedly diminished renal CXCL chemokine and CXC receptor mRNA expression in SLE mice. Renal cortical mRNA CXCL9,10,13, and 16 expression was 40–65% lower in EET-A treated SLE mice compared to vehicle treated SLE mice. Similar to chemokines, renal cortical mRNA CXCR3 and 4 receptor expression was 40–60% lower in EET-A treated SLE mice compared to vehicle treated SLE mice (Figure 1A–F).

### EET-A Treatment Reduces Renal Cytokine mRNA Expression in SLE Mice

**FIGURE 2:**
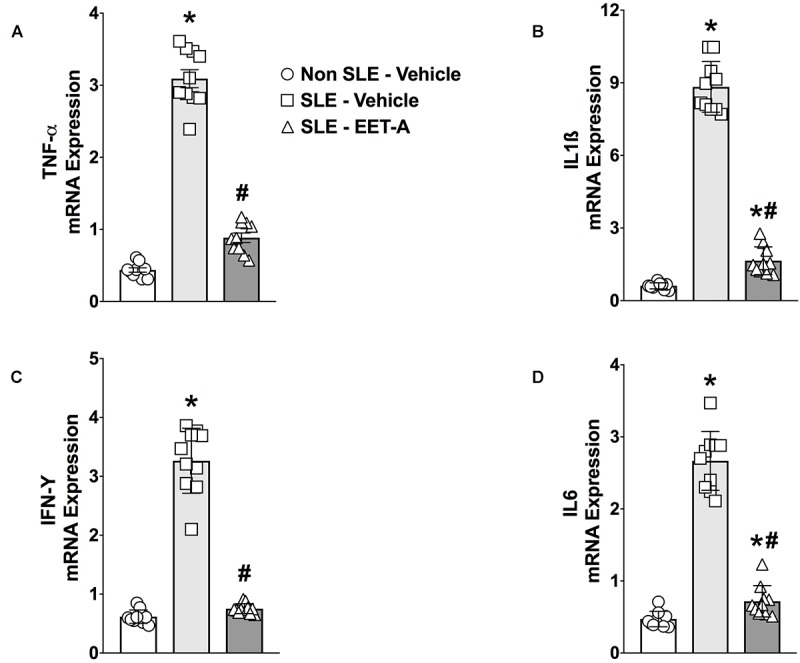
Renal cortical mRNA TNF-α **(A)**, IL-1β **(B)**, IL-6 **(C)**, and IFN-γ **(D)** expression were reduced in a mouse model of systemic lupus erythematosus (SLE). All data are expressed as Mean ± SEM, ^∗^*P* < 0.05 vs. Non SLE-Vehicle, ^#^*P* < 0.05 vs. SLE-Vehicle, *n* = 10–12/group. NZBWF1 (SLE) and NZW/LacJ (Non SLE).

Renal cortical mRNA TNF-α, IL-6, IL-1β, and IFN-γ cytokine expression was studied in the experimental groups. SLE mice had 5- to 15-fold increase in renal cortical cytokine mRNA expression compared to Non SLE mice (Figure 2A–D). EET-A treatment to SLE mice reduced renal mRNA TNF-α expression 70%, IL-6 expression 74%), IL-1β expression 80% and IFN-γ expression 76% compared to vehicle treated SLE mice (Figure 2A–D).

### Glomerular Inflammatory Cell Infiltration Decreases in EET-A Treated SLE Mice

**FIGURE 3:**
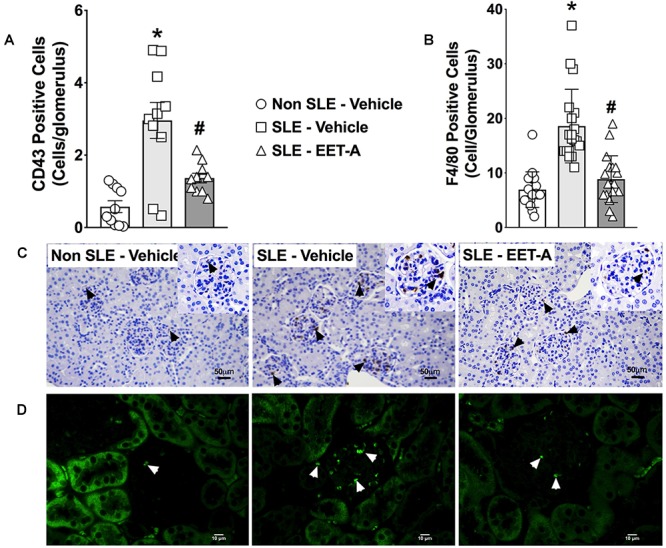
In a mouse model of systemic lupus erythematosus (SLE), EET-A reduced glomerular infiltration of CD43 positive immune cells **(A)** and F4/80 positive macrophages **(B)**. Representative photomicrographs depicting CD43 positive (black arrows) immune cells **(C)** and F4/80 positive (white arrows) macrophages **(D)** in the glomeruli for the experimental groups. All data are expressed as Mean ± SEM, ^∗^*P* < 0.05 vs. Non SLE-Vehicle, ^#^*P* < 0.05 vs. SLE-Vehicle, *n* = 10–12/group. NZBWF1 (SLE) and NZW/LacJ (Non SLE).

Glomerular inflammatory cell infiltration was markedly higher in SLE compared to Non SLE mice. SLE mice had a 5-fold increase in glomerular CD43 positive inflammatory cells compared to Non SLE mice. Like CD43 positive cells, SLE mice had a 3-fold increase in glomerular F4/80 positive inflammatory cell levels compared to Non SLE mice. Interestingly, EET-A treated SLE mice had a 50% reduction in glomerular CD43 and F4/80 positive inflammatory cells compared to vehicle treated SLE mice (Figure 3A–D).

### EET-A Treatment Decreases Albuminuria and Renal Fibrosis in SLE Mice

**FIGURE 4:**
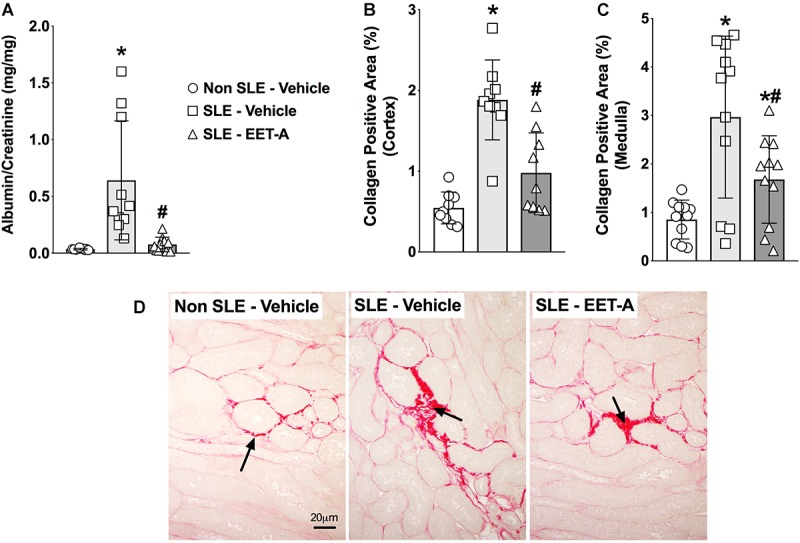
EET-A decreased albuminuria **(A)** and reduced collagen positive fibrotic area in the renal cortex **(B)** and medulla **(C)** of a mouse model of systemic lupus erythematosus (SLE). A representative photomicrograph depicting collagen positive fibrotic areas in the kidney for the experimental groups **(D)**. The black arrows showing collagen positive fibrotic areas in the kidney. All data are expressed as Mean ± SEM, ^∗^*P* < 0.05 vs. Non SLE-Vehicle, ^#^*P* < 0.05 vs. SLE-Vehicle, *n* = 10–12/group. NZBWF1 (SLE) and NZW/LacJ (Non SLE).

In the present study, SLE mice developed kidney injury a dramatic increase in albuminuria compared to Non SLE mice (Figure 4A). Interestingly, EET-A treatment to SLE mice for 14 weeks prominently attenuated renal injury in SLE mice and the albuminuria was 80% lower than vehicle treated SLE mice and at a level similar to Non SLE mice (Figure 4A). Along with albuminuria, we assessed kidney injury by measuring BUN and serum creatinine levels in the experimental groups. Serum creatinine levels were higher in vehicle treated SLE mice (5.0 ± 0.1 mg/dl, *n* = 10) compared to Non SLE mice (1.8 ± 0.1 mg/dl, *P* < 0.05, *n* = 10). Interestingly, serum creatinine levels in EET-A treated SLE mice were lower (3.8 ± 0.2 mg/dl, *P* < 0.05, *n* = 10) than vehicle treated SLE mice. SLE mice also had higher BUN levels (54.2 ± 1.7 mg/dl, *n* = 10) compared to Non SLE mice (31.3 ± 1.4 mg/dl, *P* < 0.05, *n* = 10). EET-A treated SLE mice had decreased BUN levels (46.4 ± 2.0 md/dl, *P* < 0.05, *n* = 10) compared vehicle treated SLE mice. SLE mice developed marked renal fibrosis with higher interstitial collagen formation compared to Non SLE mice. Collagen positive renal fibrotic cortical and medullary areas were 4-fold higher in vehicle treated SLR mice compared to Non SLE mice. EET-A treatment in SLE mice significantly decreased renal fibrosis (Figure 4B–D).

## Discussion

Systemic lupus erythematosus presents with a diverse array of clinical symptoms, which often reflect the consequences of injury to multiple organ systems, such as the kidney, brain, and skin. The renal manifestation of SLE which is termed LN is a major risk factor for overall morbidity and mortality in SLE. Despite the use of potent anti-inflammatory and immunosuppressive therapies, LN still progresses from chronic kidney disease to end-stage renal disease for too many patients and warrants searching for new therapeutic options (Davis et al., 1996). Mice from specific lupus-prone strains spontaneously develop SLE with renal manifestations that closely resemble LN symptoms in patients with LN. Among these mice strains, one of the most well-stablished murine LN models is the female NZBWF1 mice utilized in the current study (Perry et al., 2011). In the present study, we investigated the kidney protective actions of synthetic EET analog in female NZBWF1 SLE mice with LN. We have developed several orally active synthetic EET analogs and demonstrated their marked kidney protective actions in several renal pathologies. These studies indicate that these EET analogs exert potent kidney protective actions through strong anti-inflammatory action in the kidney (Khan et al., 2013; Hye Khan et al., 2013, 2016). In accord to these earlier findings, the present study found that the orally active EET analog, EET-A, demonstrated renal anti-inflammatory action in female NZBWF1 SLE mice that spontaneously develop LN symptoms comparable to that observed in LN patients.

In LN pathophysiology, elevated renal inflammation caused by autoantibody formation is an important event. Typically, LN pathophysiology is linked to autoantibody formation as presence of autoantibodies results in renal inflammation followed by the development of LN (Arbuckle et al., 2003). Because of their contribution in renal inflammation, autoantibodies to dsDNA (anti-dsDNA) are linked to the development of nephritis in SLE (Fenton and Rekvig, 2007), and what separates pathogenic from non-pathogenic anti-dsDNA antibodies is not clear (Zykova et al., 2007). SLE mice with LN in the present study had elevated serum anti-dsDNA compared to Non SLE mice, and EET-A treatment to SLE mice did not affect anti-dsDNA levels. On the other hand, EET-A treated SLE mice had decreased renal inflammation, decreased proteinuria, and reduced as renal fibrosis. This finding could be related to the possible presence of more non-pathogenic verses pathogenic anti-dsDNA in the SLE LN mouse model used in the present study. However, we believe this may not be the case as the SLE mice in the present study had marked renal inflammation, renal dysfunction, proteinuria, and renal fibrosis that indicate a possible contribution for pathogenic anti-dsDNA to LN pathophysiology in SLE mice. The dissociation between renal protective EET-A actions and the level of anti-dsDNA in the SLE mice could also be related to the fact that several LN models do not have elevated anti-dsDNA. In *Tlr9*-deficient MRL/+ mice that have LN, marked proteinuria and glomerular injury occurs in the absence of autoantibodies (Nickerson et al., 2017). Additionally, a neuropsychiatric SLE model, JhD/MRL/lpr mice, does not require autoantibodies to develop neuropsychiatric disease (Wen et al., 2016). Taken together, our findings provide evidence that the EET-A kidney protection in SLE LN mice does not require a reduction in autoantibodies and is associated with the ability for EET-A to decrease renal inflammation.

Progressive kidney injury in SLE results from pathogenic anti-dsDNA antibodies that deposit as immune complexes (Cameron, 1999). Immune complex formation and/or deposition in the kidney results in the synthesis of various mediators of inflammation, cellular infiltration of immune cells, proteinuria, renal fibrosis, and progressive renal failure. We demonstrated an increase in CD43 positive immune cells in the glomeruli of SLE LN mice that was attenuated in SLE LN mice treated with EET-A. CD43 positive immune cells consist of leukocytes and dendritic cells. Leukocytes and dendritic cells are important mediators to LN progression in both mouse SLE models and human SLE patients. Renal dendritic cell infiltration is associated with poor renal outcome in SLE patients (Hill et al., 2001). Immune cells express elevated levels of molecules that are necessary for their homing that increases homing to the kidney in LN (Hase et al., 2001; Yamada et al., 2002). Mechanisms by which leukocytes contribute to tissue injury include the activation of nephritogenic antibody-producing inflammatory cells and cytokine production and recruitment (Apostolidis et al., 2011). Depleting or blocking of leukocyte activation reduces LN progression SLE mouse models (Schiffer et al., 2003). Comparable to CD43 positive immune cell kidney infiltration, we demonstrated elevated F4/80 positive macrophage glomerular infiltration in the kidney of LN mice. Interestingly, EET-A treated SLE mice had markedly decrease glomerular macrophage infiltration. This is an important finding because macrophages contribute to LN pathogenesis and progression. Renal macrophage infiltration is associated with poor clinical outcomes LN patients (Hill et al., 2001). Several studies have shown that activated macrophage populations play an important role in the pathophysiology of LN in murine models (Schiffer et al., 2008). The contribution for macrophages in LN pathophysiology is further supported by macrophage depletion studies. These studies elegantly demonstrated that macrophages are not only present during LN but also actively contribute to the LN pathogenesis (Hasegawa et al., 2003; Shimizu et al., 2004). Findings in the current study demonstrate that EET-A treated SLE mice had decreased CD43 positive immune cell and F4/F80 positive macrophage glomerular infiltration that contributes to decreased LN pathogenesis.

Renal chemokine production during LN precedes inflammatory cell infiltration, proteinuria, and renal fibrosis (Fan et al., 1997). Chemokines promote chemotaxis and activation of selected inflammatory cell subpopulations that express specific chemokine receptors (Premack and Schall, 1996). Likewise, a role for several chemokines in several acute inflammatory renal disorders including SLE has been described (Feng et al., 1995, 1999; Wenzel et al., 1997). Chemokines play an important pathophysiological role in LN. Indeed, a common pathological finding in LN is inflammatory cell infiltration in the affected tissue resulting from chemotaxis by chemokines and their receptors. In the present study, we demonstrated a marked increase renal cortical expression of CXC chemokines and their receptors in SLE mice. SLE mice with LN had higher CXCR3 and its ligands CXCL9 and 10 renal expression. Previous studies demonstrated that the CXCR3 receptor–ligand system is involved in chemotaxis of inflammatory cells in the affected target tissue. Evidence for the CXCR3 pathway in LN comes from the results of an extensive glomerular expression microarray analysis SLE MRL/lpr mice (Teramoto et al., 2008). Indeed, chemokines and chemokine receptors including CXCR3 and its ligands, CXCL9 and 10 play an important role in the pathogenesis of LN. Interestingly, prednisolone treatment to SLE MRL/lpr mice attenuated CXCR3 receptor upregulation inflammatory cell infiltration (Lacotte et al., 2009). In the present study, along with higher CXCR3 receptor-ligand system expression, there was marked glomerular CD43 immune cell and F4/F80 macrophage infiltration in the kidney of LN mice. This finding corroborates earlier findings on the pathophysiological role for CXCR3 and its ligands in LN. Interestingly, SLE mice treated with EET-A had markedly lower renal cortical CXCR3 receptor–ligand mRNA expression. EET-A treated SLE LN mice also had fewer CD43 immune cells and F4/F80 macrophages in the glomeruli. These findings clearly indicate a strong ability for EET-A to attenuate chemotaxis and glomerular inflammatory cell infiltration in LN.

The chemotaxis attenuating effect of EET-A was further evident from its ability to decrease several other chemokines and receptors that contribute to LN pathophysiology. We demonstrate marked renal CXCR4 expression in SLE mice that is in agreement with earlier findings indicated that CXCR4 is crucial for SLE pathogenesis in mice (Wang et al., 2009). Elevated CXCR4 levels in SLE mice were found to prolong inflammatory cell migration to end-organs via its ligand (Wang et al., 2009). In addition, CXCR4 ligand has been shown to be selectively upregulated in kidney glomeruli of NZB/W, BXSB, and MRL/lpr SLE mice models with LN and in LN patients. (Balabanian et al., 2003; Togel et al., 2005; Chong and Mohan, 2009; Wang et al., 2010). The current findings in SLE LN mice also demonstrate a contribution for the CXC chemokines CXCL13 and CXCl16. Moreover, EET-A treated SLE mice had reduced renal mRNA expression of these important chemokines that are implicated in LN pathophysiology. CXCL13 is an important chemokine for LN pathology and several human and animal studies support its critical role in LN. Serum CXCL13 levels and kidney CXCL13 mRNA expression have been found to be higher in LN patients with increased disease severity (Lee et al., 2010; Ezzat et al., 2011). Likewise, increased CXCL13 expression has been demonstrated in female NZBWF1 LN mice (Shen et al., 2012). Our findings also demonstrate that SLE LN mice had markedly higher renal cortical CXCL16 mRNA expression that is a key mediator of the renal inflammation in LN (Norlander et al., 2013). CXCL16 has been shown to be elevated in several strains of mice and patients with LN and correlates well with elevated proteinuria and SLE disease activity index scores (Wu et al., 2007). Overall, our findings corroborate several earlier findings that clearly indicated a critical role for chemokines in LN. Most importantly, we determined that an orally active EET analog, EET-A, prevented LN in SLE mice in part by preventing chemotaxis and glomerular inflammatory cell infiltration in the kidney.

Although it is apparent that chemokines contribute significantly to inflammatory cell influx into sites of tissue injury, chemokines must be considered part of a concerted interaction involving cytokines. Indeed, renal upregulation of several proinflammatory cytokines, such as TNF-α, IFN-γ, and IL-1β was observed in SLE LN mice. These proinflammatory cytokines have been demonstrated to participate in several pathophysiologic processes in SLE mice. For example, IFN-γ is required for LN. IFN-γ deficient MRL/lpr mice are protected from lymphadenopathy and early death, and the severity of renal damage was reduced in these mice (Balomenos et al., 1998; Schwarting et al., 1998). In kidney biopsies from LN patients, TNF-α and IL-6 mRNA expressions were elevated (Herrera-Esparza et al., 1998). In addition, administration of anti-TNF-α antibodies was able to abrogate mercuric chloride-induced lupus-like autoimmune disease in rats (Molina et al., 1995). In our study, involvement of cytokines in LN pathophysiology was associated with elevated renal mRNA TNF-α, IL-6, IL-1β, and IFN-γ cytokine expression in female NZBWF1 SLE mice. Indeed, these findings clearly corroborate several earlier findings on cytokine contribution to LN pathophysiology (Balomenos et al., 1998; Herrera-Esparza et al., 1998; Schwarting et al., 1998). As an important finding, EET-A treatment decreased mRNA TNF-α, IL-6, IL-1β, and IFN-γ expression in the kidney cortex of LN mice. This finding further strengthens our findings that EET-A has strong renal anti-inflammatory action to protect the kidney in SLE from LN.

In summary, the current study demonstrates elevated renal chemotaxis that can result in cytokine production and renal glomeruli inflammatory cell infiltration in female NZBWF1 SLE LN mice. These findings clearly indicate potent EET-A kidney protective and anti-inflammatory actions in SLE LN mice which is associated with decreased chemotaxis and cytokine expression in the kidney. Most importantly, we provide unique data on the biological actions for a synthetic EET analog and the potential for an EET analog-based novel SLE LN therapy.

## Author Contributions

MAHK and JI conceived the study, interpreted the data, and wrote the manuscript. MAHK and AS performed the experiments. JF and MAS designed and synthesized EET-A. All authors edited the manuscript and approved the submitted version.

## Conflict of Interest Statement

JI and JF have patents that covers the composition of matter for EET-A. The remaining authors declare that the research was conducted in the absence of any commercial or financial relationships that could be construed as a potential conflict of interest.

## References

[B1] Apostolidis S. A. Crispin J. C. Tsokos G. C. (2011). IL-17-producing T cells in lupus nephritis. *Lupus* 20 120–124. 10.1177/0961203310389100 21303828

[B2] Arbuckle M. R. McClain M. T. Rubertone M. V. Scofield R. H. Dennis G. J. James J. A. (2003). Development of autoantibodies before the clinical onset of systemic lupus erythematosus. *N. Engl. J. Med.* 349 1526–1533. 14561795 10.1056/NEJMoa021933

[B3] Balabanian K. Couderc J. Bouchet-Delbos L. Amara A. Berrebi D. Foussat A. (2003). Role of the chemokine stromal cell-derived factor 1 in autoantibody production and nephritis in murine lupus. *J. Immunol.* 170 3392–3400. 10.4049/jimmunol.170.6.3392 12626600

[B4] Balomenos D. Rumold R. Theofilopoulos A. N. (1998). Interferon gamma is required for lupus-like disease and lympho accumulation in MRL-lpr mice. *J. Clin. Invest.* 101 364–371. 10.1172/jci750 9435308 PMC508575

[B5] Cameron J. S. (1999). Lupus nephritis. *J. Am. Soc. Nephrol.* 10 413–424. 10215343 10.1681/ASN.V102413

[B6] Chong B. F. Mohan C. (2009). Targeting the CXCR4/CXCL12 axis in systemic lupus erythematosus. *Expert. Opin. Ther. Targets* 13 1147–1153. 10.1517/14728220903196761 19670960

[B7] Contreras G. Pardo V. Leclercq B. Lenz O. Tozman E. O’Nan P. (2004). Sequential therapies for proliferative lupus nephritis. *N. Engl. J. Med.* 350 971–980. 10.1056/nejmoa031855 14999109

[B8] Dall’Era M. (2017). Treatment of lupus nephritis: current paradigms and emerging strategies. *Curr. Opin. Rheumatol.* 29 241–247. 10.1097/BOR.0000000000000381 28207493

[B9] Davis J. C. Tassiulas I. O. Boumpas D. T. (1996). Lupus nephritis. *Curr. Opin. Rheumatol.* 8 415–423. 8941444 10.1097/00002281-199609000-00005

[B10] Ezzat M. El-Gammasy T. Shaheen K. Shokr E. (2011). Elevated production of serum B-cell-attracting chemokine-1 (BCA-1/CXCL13) is correlated with childhood-onset lupus disease activity, severity, and renal involvement. *Lupus* 20 845–854. 10.1177/0961203311398513 21576203

[B11] Fan X. Oertli B. Wuthrich R. P. (1997). Up-regulation of tubular epithelial interleukin-12 in autoimmune MRL-Fas(lpr) mice with renal injury. *Kidney Int.* 51 79–86. 10.1038/ki.1997.10 8995720

[B12] Feng L. Chen S. Garcia G. E. Xia Y. Siani M. A. Botti P. (1999). Prevention of crescentic glomerulonephritis by immunoneutralization of the fractalkine receptor CX3CR1: rapid communication. *Kidney Int.* 56 612–620. 10.1046/j.1523-1755.1999.00604.x 10432400

[B13] Feng L. Xia Y. Yoshimura T. Wilson C. B. (1995). Modulation of neutrophil influx in glomerulonephritis in the rat with anti-macrophage inflammatory protein-2 (MIP-2) antibody. *J. Clin. Invest.* 95 1009–1017. 10.1172/jci117745 7883948 PMC441434

[B14] Fenton K. A. Rekvig O. P. (2007). A central role of nucleosomes in lupus nephritis. *Ann. N. Y. Acad. Sci.* 1108 104–113. 10.1196/annals.1422.012 17893976

[B15] Gilkeson G. S. (2015). Complement-targeted therapies in Lupus. *Curr. Treat. Options Rheum.* 1 10–18. 10.1016/j.ekir.2016.06.005 29142924 PMC5678788

[B16] Hase K. Tani K. Shimizu T. Ohmoto Y. Matsushima K. Sone S. (2001). Increased CCR4 expression in active systemic lupus erythematosus. *J. Leukoc. Biol.* 70 749–755. 11698495

[B17] Hasegawa H. Kohno M. Sasaki M. Inoue A. Ito M. R. Terada M. (2003). Antagonist of monocyte chemoattractant protein 1 ameliorates the initiation and progression of lupus nephritis and renal vasculitis in MRL/lpr mice. *Arthritis Rheum* 48 2555–2566. 10.1002/art.11231 13130475

[B18] Herrera-Esparza R. Barbosa-Cisneros O. Villalobos-Hurtado R. Avalos-Diaz E. (1998). Renal expression of IL-6 and TNFalpha genes in lupus nephritis. *Lupus* 7 154–158. 10.1191/096120398678919949 9607638

[B19] Hill G. S. Delahousse M. Nochy D. Mandet C. Bariety J. (2001). Proteinuria and tubulointerstitial lesions in lupus nephritis. *Kidney Int.* 60 1893–1903. 10.1046/j.1523-1755.2001.00017.x 11703608

[B20] Hye Khan M. A. Fish B. Wahl G. Sharma A. Falck J. R. Paudyal M. P. (2016). Epoxyeicosatrienoic acid analogue mitigates kidney injury in a rat model of radiation nephropathy. *Clin. Sci.* 130 587–599. 10.1042/CS20150778 26772189 PMC5020909

[B21] Hye Khan M. A. Kolb L. Skibba M. Hartmann M. Blöcher R. Proschak E. (2018). A novel dual PPAR-γ agonist/sEH inhibitor treats diabetic complications in a rat model of type 2 diabetes. *Diabetologia* 61 2235–2246. 10.1007/s00125-018-4685-0 30032428 PMC6563928

[B22] Hye Khan M. A. Neckár J. Manthati V. Errabelli R. Pavlov T. S. Staruschenko A. (2013). Orally active epoxyeicosatrienoic acid analog attenuates kidney injury in hypertensive Dahl salt-sensitive rat. *Hypertension* 62 905–913. 10.1161/HYPERTENSIONAHA.113.01949 23980070 PMC3872985

[B23] Jordan N. D’Cruz D. (2016). Current and emerging treatment options in the management of lupus. *Immunotargets Ther.* 5 9–20. 10.2147/ITT.S40675 27529058 PMC4970629

[B24] Khan M. A. Liu J. Kumar G. Skapek S. X. Falck J. R. Imig J. D. (2013). Novel orally active epoxyeicosatrienoic acid (EET) analogs attenuate cisplatin nephrotoxicity. *FASEB J.* 27 2946–2956. 10.1096/fj.12-218040 23603837 PMC3714587

[B25] Lacotte S. Brun S. Muller S. Dumortier H. (2009). CXCR3, inflammation, and autoimmune diseases. *Ann. N. Y. Acad. Sci.* 1173 310–317. 10.1111/j.1749-6632.2009.04813.x 19758167

[B26] Lech M. Anders H. J. (2013). The pathogenesis of lupus nephritis. *J. Am. Soc. Nephrol.* 24 1357–1366. 10.1681/ASN.2013010026 23929771 PMC3752952

[B27] Lee H. T. Shiao Y. M. Wu T. H. Chen W. S. Hsu Y. H. Tsai S. F. (2010). Serum BLC/CXCL13 concentrations and renal expression of CXCL13/CXCR5 in patients with systemic lupus erythematosus and lupus nephritis. *J. Rheumatol.* 37 45–52. 10.3899/jrheum.090450 19955043

[B28] Lu J. Szeto C. C. Tam L. S. Lai F. M. Li E. K. Chow K. M. (2012). Relationship of intrarenal gene expression and the histological class of lupus nephritis – a study on repeat renal biopsy. *J. Rheumatol.* 39 1942–1947. 10.3899/jrheum.120177 22896023

[B29] Molina A. Sa′nchez-Madrid F. Bricio T. Mart′n A. Escudero E. Alvarez V. (1995). Abrogation of mercuric chloride induced nephritis in the Brown Norway rat by treatment with antibodies against TNFalpha. *Med. Inflamm.* 4 444–451. 10.1155/s0962935195000718 18475678 PMC2365666

[B30] Nickerson K. M. Wang Y. Bastacky S. Shlomchik M. J. (2017). Toll-like receptor 9 suppresses lupus disease in Fas-sufficient MRL Mice. *PLoS One* 12:e0173471. 10.1371/journal.pone.0173471 28278279 PMC5344451

[B31] Norlander A. E. Saleh M. A. Madhur M. S. (2013). CXCL16: a chemokine-causing chronic kidney disease. *Hypertension* 62 1008–1010. 10.1161/hypertensionaha.113.01954 24060889 PMC3970762

[B32] Perry D. Sang A. Yin Y. Zheng Y. Y. Morel L. (2011). Murine models of systemic lupus erythematosus. *J. Biomed. Biotechnol.* 2011:271694. 10.1155/2011/271694 21403825 PMC3042628

[B33] Premack B. A. Schall T. J. (1996). Chemokine receptors: gateways to inflammation and infection. *Nat. Med.* 2 1174–1178. 10.1038/nm1196-1174 8898734

[B34] Schiffer L. Bethunaickan R. Ramanujam M. Huang W. Schiffer M. Tao H. (2008). Activated renal macrophages are markers of disease onset and disease remission in lupus nephritis. *J. Immunol.* 180 1938–1947. 10.4049/jimmunol.180.3.1938 18209092 PMC2587994

[B35] Schiffer L. Sinha J. Wang X. Huang W. von Gersdorff G. Schiffer M. (2003). Short term administration of costimulatory blockade and cyclophosphamide induces remission of systemic lupus erythematosus nephritis in NZB/W F1 mice by a mechanism downstream of renal immune complex deposition. *J. Immunol.* 171 489–497. 10.4049/jimmunol.171.1.489 12817034

[B36] Schwarting A. Wada T. Kinoshita K. Tesch G. Kelley V. R. (1998). IFN-g receptor signaling is essential for the initiation, acceleration, and destruction of autoimmune kidney disease in MRL-Fas(lpr) mice. *J. Immunol.* 161 494–503. 9647261

[B37] Shen Y. Sun C. Y. Wu F. X. Chen Y. Dai M. Yan Y. C. (2012). Association of intrarenal B-cell infiltrates with clinical outcome in lupus nephritis: a study of 192 cases. *Clin. Dev. Immunol* 2012:967584. 10.1155/2012/967584 22792121 PMC3389683

[B38] Shimizu S. Nakashima H. Masutani K. Inoue Y. Miyake K. Akahoshi M. (2004). Anti-monocyte chemoattractant protein-1 gene therapy attenuates nephritis in MRL/lpr mice. *Rheumatology* 43 1121–1128. 10.1093/rheumatology/keh277 15213333

[B39] Skibba M. Hye Khan M. A. Kolb L. L. Yeboah M. M. Falck J. R. Amaradhi R. (2017). Epoxyeicosatrienoic acid analog decreases renal fibrosis by reducing epithelial-to-mesenchymal transition. *Front. Pharmacol.* 8:406. 10.3389/fphar.2017.00406 28713267 PMC5491687

[B40] Teramoto K. Negoro N. Kitamoto K. Iwai T. Iwao H. Okamura M. (2008). Microarray analysis of glomerular gene expression in murine lupus nephritis. *J. Pharmacol. Sci.* 106 56–67. 10.1254/jphs.fp0071337 18187931

[B41] Togel F. Isaac J. Hu Z. Weiss K. Westenfelder C. (2005). Renal SDF-1 signals mobilization and homing of CXCR4-positive cells to the kidney after ischemic injury. *Kidney Int.* 67 1772–1784. 10.1111/j.1523-1755.2005.00275.x 15840024

[B42] Wang A. Fairhurst A. M. Tus K. Subramanian S. Liu Y. Lin F. (2009). CXCR4/CXCL12 hyperexpression plays a pivotal role in the pathogenesis of lupus. *J. Immunol.* 182 4448–4458. 10.4049/jimmunol.0801920 19299746 PMC2946082

[B43] Wang A. Guilpain P. Chong B. F. Chouzenoux S. Guillevin L. Du Y. (2010). Dysregulated expression of CXCR4/CXCL12 in subsets of patients with systemic lupus erythematosus. *Arthritis Rheum* 2010 3436–3446. 10.1002/art.27685 20722038 PMC8972909

[B44] Wen J. Doerner J. Chalmers S. Stock A. Wang H. Gullinello M. (2016). B cell and/or autoantibody deficiency do not prevent neuropsychiatric disease in murine systemic lupus erythematosus. *J. Neuroinflamm.* 13:73. 10.1186/s12974-016-0537-3 PMC4823887 27055816

[B45] Wenzel U. Schneider A. Valente A. J. Abboud H. E. Thaiss F. Helmchen U. M. (1997). Monocyte chemoattractant protein-1 mediates monocyte/macrophage influx in anti-thymocyte antibody- induced glomerulonephritis. *Kidney Int.* 51 770–776. 10.1038/ki.1997.108 9067909

[B46] Wu T. Xie C. Wang H. W. Zhou X. J. Schwartz N. Calixto S. (2007). Elevated urinary vcam-1, p-selectin, soluble tnf receptor-1, and cxc chemokine ligand 16 in multiple murine lupus strains and human lupus nephritis. *J. Immunol.* 179 7166–7175. 10.4049/jimmunol.179.10.7166 17982109

[B47] Yamada M. Yagita H. Inoue H. Takanashi T. Matsuda H. Munechika E. (2002). Selective accumulation of CCR4+ T lymphocytes into renal tissue of patients with lupus nephritis. *Arthritis Rheum* 46 735–740. 10.1002/art.10112 11920409

[B48] Yeboah M. M. Hye Khan M. A. Chesnik M. A. Sharma A. Paudyal M. P. Falck J. R. (2016). The epoxyeicosatrienoic acid analog PVPA ameliorates cyclosporine-induced hypertension and renal injury in rats. *Am. J. Physiol. Renal Physiol.* 311 F576–F585. 10.1152/ajprenal.00288.2016 27358055 PMC5504406

[B49] Zykova S. N. Seredkina N. E. Rekvig O. P. (2007). Glomerular targets for autoantibodies in lupus nephritis – an apoptotic origin. *Ann. N. Y. Acad. Sci.* 1108 1–10. 10.1196/annals.1422.001 17893965

